# Supplementary material for: Tumor-specific lncRNA IGF1R-AS1 trans-regulates chromatin interactions associated with oncogenic MYC signaling
Source: Nat Commun. 2026 Mar 19;17:4171. doi: 10.1038/s41467-026-70814-4 (PMC13153188; doi:10.1038/s41467-026-70814-4)
Supplement: Supplementary file 1 — Supplementary Information [file 41467_2026_70814_MOESM1_ESM.pdf]

Supplemental information

Tumor-specific lncRNA *IGF1R-AS1* trans-regulates chromatin interactions associated with oncogenic MYC signaling

Yongyong Yang[1,*], Ting-You Wang[1,*], Joshua Fry[1,2,*], Yingming Li[3,*], Qingshu Meng[1,*], Qingxiang Guo[1], Nathan E. Patchen[4], Kyle H. White[5,†], Abhirami Ramakrishnan[1], Yanan Ren[1], Qianru Li[6], Xingxing Zhang[1], Taufeeque Ali[1], Courtney Dawes[4], Stamatina Fragkogianni[7], Parker Irvin[5], Sk Kayum Alam[8], Luke H. Hoeppner[3,8], Xihong Zhang[3], Douglas Yee[3], Adam B. Weiner[9], Edward M. Schaeffer[1], Yang Liu[4], Xiaoyang Zhang[5], Scott M. Dehm[3,10,$], Qi Cao[1,11,$], Rendong Yang[1,11,$]

[1] Department of Urology, Northwestern University Feinberg School of Medicine, Chicago, IL 60611, USA.
[2] Bioinformatics and Computational Biology Program, University of Minnesota, Minneapolis, MN 55455, USA.
[3] Masonic Cancer Center, University of Minnesota, Minneapolis, MN 55455, USA.
[4] Department of Biochemistry, University of Utah, Salt Lake City, UT 84112, USA.
[5] Department of Oncological Sciences, Huntsman Cancer Institute, University of Utah, Salt Lake City, UT 84112, USA.
[6] Department of Pharmacology, Feinberg School of Medicine, Northwestern University, Chicago, IL 60611, USA.
[7] Tempus AI, Inc., Chicago, IL 60654, USA.
[8] The Hormel Institute, University of Minnesota, Austin, MN 55912, USA.
[9] Department of Urology, Cedars Sinai Medical Center, Los Angeles, CA 90048, USA.
[10] Departments of Laboratory Medicine and Pathology and Urology, University of Minnesota, Minneapolis, MN 55455, USA.
[11] Robert H. Lurie Comprehensive Cancer Center, Northwestern University Feinberg School of Medicine, Chicago, IL 60611, USA.
[†] Present address: Cancer Research Program, Research Institute of the McGill University Health Centre, Montreal, QC H4A 3J1, Canada.
[*] Equal contribution
[$] Corresponding authors

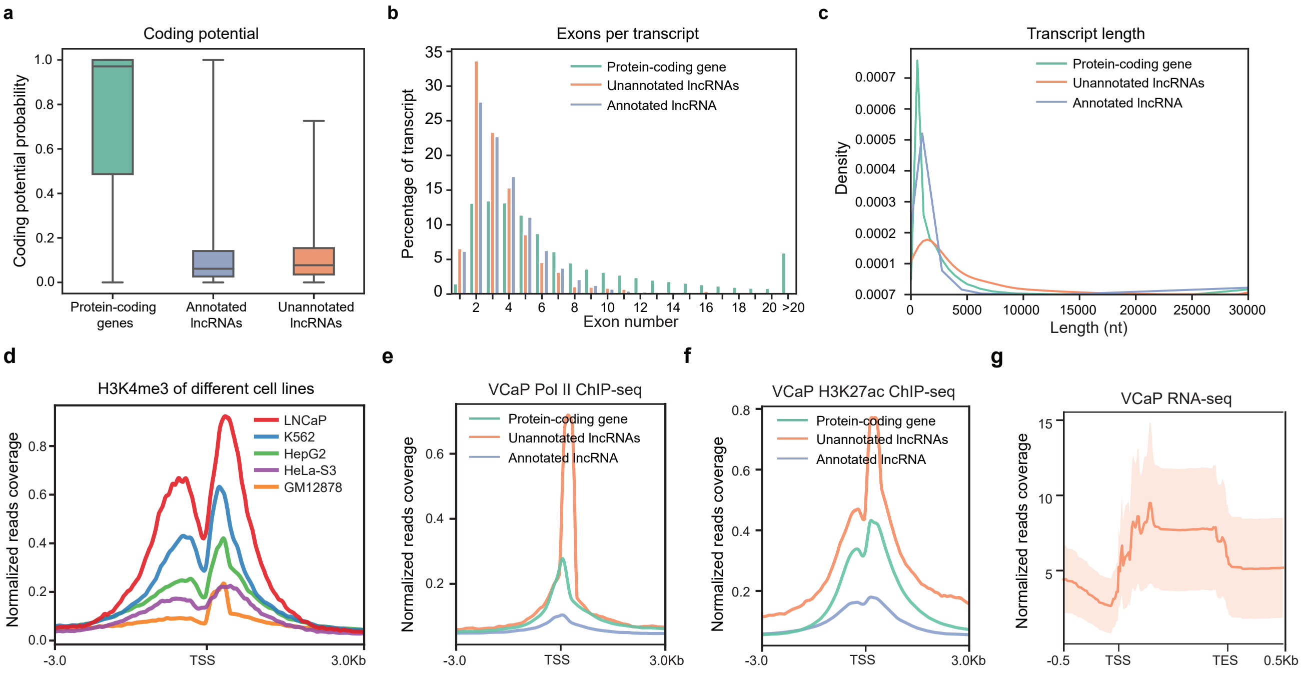

a
Coding potential
Coding potential probability
Protein-coding genes
Annotated lncRNAs
Unannotated lncRNAs
b
Exons per transcript
Percentage of transcript
Exon number
Protein-coding gene
Unannotated lncRNAs
Annotated lncRNA
c
Transcript length
Density
Length (nt)
Protein-coding gene
Unannotated lncRNAs
Annotated lncRNA
d
H3K4me3 of different cell lines
Normalized reads coverage
LNCaP
K562
HepG2
HeLa-S3
GM12878
-3.0
TSS
3.0Kb
e
VCaP Pol II ChIP-seq
Normalized reads coverage
Protein-coding gene
Unannotated lncRNAs
Annotated lncRNA
f
VCaP H3K27ac ChIP-seq
Normalized reads coverage
g
VCaP RNA-seq
Normalized reads coverage
-0.5
TSS
TES
0.5Kb

Supplementary Figure 1. Characterization of lncRNAs in mCRPC. (a) Predicted coding-potential of protein-coding genes (n=19,951), annotated lncRNAs (n=16,892), and unannotated lncRNAs (n=1,344). **(b)** Distribution of number of exons per transcript of protein-coding genes (n=19,951), annotated lncRNAs (n=16,892), and unannotated lncRNAs (n=1,344). **(c)** Distribution of transcript lengths for protein-coding genes (n=19,951), annotated lncRNAs (n=16,892), and unannotated lncRNAs (n=1,344). **(d)** Unannotated lncRNA associated active promoter histone signal H3K4me3 in different cancer cell lines (n=1,344). **(e-f)** Plots displaying the enrichment of Pol II (e) and H3K27ac (f) ChIP-seq signals at the TSS of protein-coding genes (n=19,951), annotated lncRNAs (n=16,892), and unannotated lncRNAs (n=1,344) in VCaP cells. **(g)** Aggregate RNA-seq read density along the pseudo-length of unannotated lncRNA genes in VCaP prostate cancer cells. The plot visualizes the normalized RNA-seq read coverage from 0.5 kb upstream of the transcription start site (TSS) to 0.5 kb downstream of the transcription end site (TES) for all unannotated lncRNA genes (n=1,344). The solid orange line represents the mean normalized read coverage, while the shaded region indicates the standard deviation across genes. This profile highlights consistent transcriptional activity across the gene body.

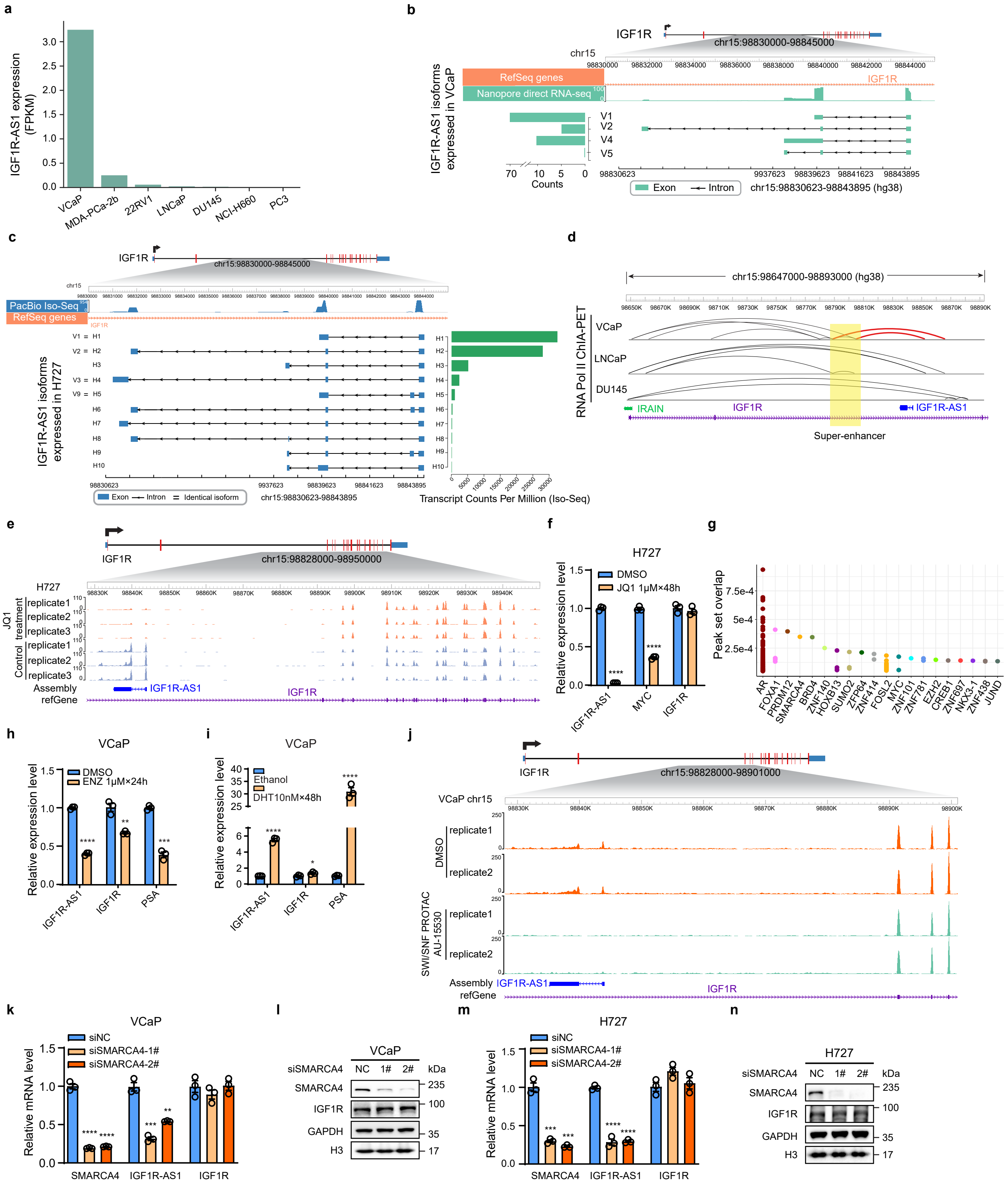
a
IGF1R-AS1 expression (FPKM)
VCaP
MDA-PCa-2b
22RV1
LNCaP
DU145
NCI-H660
PC3
b
IGF1R
chr15:98830000-98845000
chr15
RefSeq genes
Nanopore direct RNA-seq
IGF1R-AS1 isoforms expressed in VCaP
V1
V2
V4
V5
Counts
Exon
Intron
chr15:98830623-98843895 (hg38)
c
IGF1R
chr15:98830000-98845000
PacBio Iso-Seq
RefSeq genes
IGF1R-AS1 isoforms expressed in H727
V1 = H1
V2 = H2
H3
V3 = H4
V9 = H5
H6
H7
H8
H9
H10
Exon
Intron
= Identical isoform
chr15:98830623-98843895
Transcript Counts Per Million (Iso-Seq)
d
chr15:98647000-98893000 (hg38)
RNA Pol II ChIA-PET
VCaP
LNCaP
DU145
IRAIN
IGF1R
IGF1R-AS1
Super-enhancer
e
IGF1R
chr15:98828000-98950000
H727
JQ1 treatment
Control
replicate1
replicate2
replicate3
Assembly
refGene
IGF1R-AS1
IGF1R
f
H727
DMSO
JQ1 1μM×48h
Relative expression level
IGF1R-AS1
MYC
IGF1R
g
Peak set overlap
AR
FOXA1
PRDM12
SMARCA4
BRD4
ZNF140
HOXB13
SUMO2
ZFP64
ZNF414
FOSL2
MYC
ZNF101
ZNF781
EZH2
CREB1
ZNF697
NKX3-1
ZNF438
JUND
h
VCaP
DMSO
ENZ 1μM×24h
Relative expression level
IGF1R-AS1
IGF1R
PSA
i
VCaP
Ethanol
DHT10nM×48h
Relative expression level
IGF1R-AS1
IGF1R
PSA
j
IGF1R
chr15:98828000-98901000
VCaP chr15
DMSO
SW/SNF PROTAC AU-15530
replicate1
replicate2
Assembly
refGene
IGF1R-AS1
IGF1R
k
VCaP
siNC
siSMARCA4-1#
siSMARCA4-2#
Relative mRNA level
SMARCA4
IGF1R-AS1
IGF1R
l
VCaP
siSMARCA4
NC
1#
2#
kDa
SMARCA4
IGF1R
GAPDH
H3
m
H727
siNC
siSMARCA4-1#
siSMARCA4-2#
Relative mRNA level
SMARCA4
IGF1R-AS1
IGF1R
n
H727
siSMARCA4
NC
1#
2#
kDa
SMARCA4
IGF1R
GAPDH
H3

Supplementary Figure 2. Characterization of *IGF1R-AS1* and its regulation by SMARCA4. (a) *IGF1R-AS1* expression across prostate cancer cell lines measured by RNA-seq. **(b)** The *IGF1R-AS1* isoforms identified by Nanopore direct RNA sequencing in VCaP cells (V1, V2, V4 and V5) are the same as isoforms detected by PacBio Iso-seq in VCaP cells. **(c)** Isoforms of *IGF1R-AS1* identified by PacBio long-read sequencing in H727 cells include ten distinct isoforms (H1 – H10). Among these, four isoforms (H1, H2, H4, and H5) are identical to those discovered in VCaP cells (V1, V2, V3 and V9). **(d)** Chromatin interactions indicated by RNA PolII ChIA-PET in different prostate cancer cell lines. Red curve indicates the interactions between *IGF1R-AS1* promoter and its associated super-enhancer. **(e)** RNA-seq expression tracks show *IGF1R-AS1* and *IGF1R* expression under JQ1 treatment and control conditions in H727 cells. **(f)** qPCR analysis of expression of *IGF1R-AS1*, *IGF1R*, and the known super-enhancer target gene *MYC* in H727 cells treated with JQ1 and DMSO (n= 3 biological replicates). Unpaired Student's t-test, two-sided. p****<0.0001. **(g)** Transcription factors that bind to the *IGF1R-AS1* super-enhancer region (chr15:98802509-98805287, hg38) revealed by CistromeDB Toolkit (http://dbtoolkit.cistrome.org/). **(h)** qPCR analysis of *IGF1R-AS1*, *IGF1R* and *PSA* expression in VCaP cells with AR inhibition by Enzalutamide (ENZ) treatment (n = 3 biological replicates). Unpaired Student's t-test, two-sided. p**=0.0031, p***=0.0001, p****<0.0001. **(i)** qPCR analysis of *IGF1R-AS1*, *IGF1R* and *PSA* in VCaP cells with dihydrotestosterone (DHT) treatment (n=3 biological replicates). Unpaired Student's t-test, two-sided. p*=0.0415, p****<0.0001. **(j)** RNA-seq expression tracks show *IGF1R-AS1* and *IGF1R* expression in SWI/SNF PROTAC degrader AU-15530 and DMSO treated in VCaP cells. RNA-seq were obtained from GEO with access code GSE171592. **(k)** qPCR analysis of *SMARCA4*, *IGF1R-AS1*, and *IGF1R* expression in VCaP cells after *SMARCA4* knockdown (n=3 biological replicates). Unpaired Student's t-test, two-sided. p**=0.0011, p***=0.0003, p****<0.0001. **(l)** Western blotting analysis of SMARCA4 and IGF1R expression in VCaP cells after *SMARCA4* knockdown. **(m)** qPCR analysis of *SMARCA4*, *IGF1R-AS1*, and *IGF1R* expression in H727 cells after *SMARCA4* knockdown (n=3 biological replicates). Unpaired Student's t-test, two-sided. p***(*SMARCA4*, siNC vs siSMARCA4-1#)=0.0003, p***(*SMARCA4*, siNC vs siSMARCA4-2#)=0.0002, p****<0.0001. **(n)** Western blotting analysis of SMARCA4 and IGF1R expression in H727 cells after *SMARCA4* knockdown. Data in (f, h-i, k, and m) are shown as mean ± SEM. Experiments in (l and n) were biologically repeated three times. Source data of this Figure are provided as Source data file.

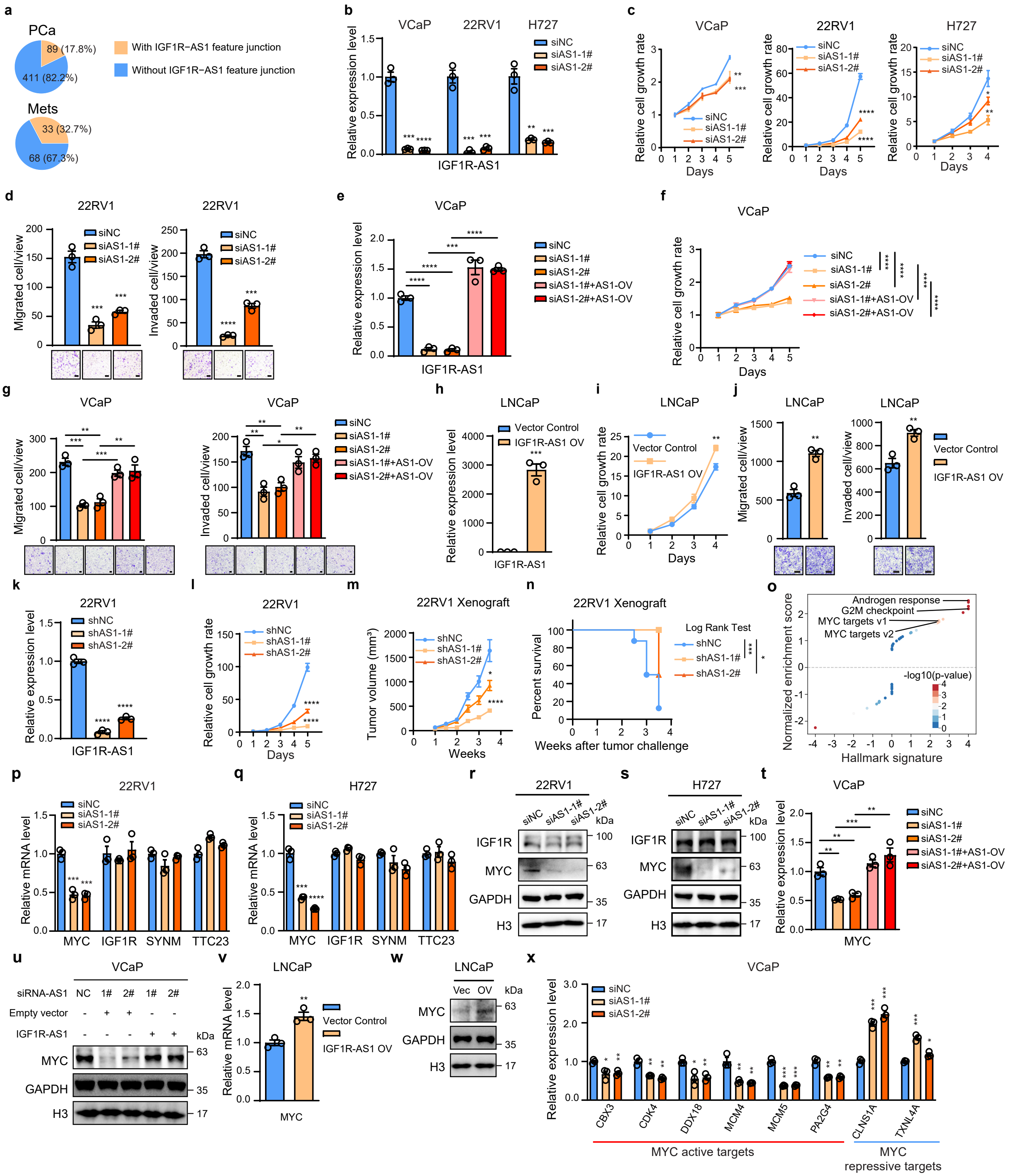
a
PCa
89 (17.8%)
411 (82.2%)
With IGF1R−AS1 feature junction
Without IGF1R−AS1 feature junction
Mets
33 (32.7%)
68 (67.3%)
b
VCaP
22RV1
H727
siNC
siAS1-1#
siAS1-2#
Relative expression level
IGF1R-AS1
c
VCaP
22RV1
H727
Relative cell growth rate
Days
siNC
siAS1-1#
siAS1-2#
d
22RV1
22RV1
Migrated cell/view
Invaded cell/view
siNC
siAS1-1#
siAS1-2#
e
VCaP
Relative expression level
IGF1R-AS1
siNC
siAS1-1#
siAS1-2#
siAS1-1#+AS1-OV
siAS1-2#+AS1-OV
f
VCaP
Relative cell growth rate
Days
siNC
siAS1-1#
siAS1-2#
siAS1-1#+AS1-OV
siAS1-2#+AS1-OV
g
VCaP
VCaP
Migrated cell/view
Invaded cell/view
siNC
siAS1-1#
siAS1-2#
siAS1-1#+AS1-OV
siAS1-2#+AS1-OV
h
LNCaP
Vector Control
IGF1R-AS1 OV
Relative expression level
IGF1R-AS1
i
LNCaP
Vector Control
IGF1R-AS1 OV
Relative cell growth rate
Days
j
LNCaP
LNCaP
Migrated cell/view
Invaded cell/view
Vector Control
IGF1R-AS1 OV
k
22RV1
shNC
shAS1-1#
shAS1-2#
Relative expression level
IGF1R-AS1
l
22RV1
shNC
shAS1-1#
shAS1-2#
Relative cell growth rate
Days
m
22RV1 Xenograft
shNC
shAS1-1#
shAS1-2#
Tumor volume (mm³)
Weeks
n
22RV1 Xenograft
Log Rank Test
shNC
shAS1-1#
shAS1-2#
Percent survival
Weeks after tumor challenge
o
Androgen response
G2M checkpoint
MYC targets v1
MYC targets v2
-log10(p-value)
Normalized enrichment score
Hallmark signature
p
22RV1
siNC
siAS1-1#
siAS1-2#
Relative mRNA level
MYC
IGF1R
SYNM
TTC23
q
H727
siNC
siAS1-1#
siAS1-2#
Relative mRNA level
MYC
IGF1R
SYNM
TTC23
r
22RV1
siNC
siAS1-1#
siAS1-2#
kDa
IGF1R
MYC
GAPDH
H3
s
H727
siNC
siAS1-1#
siAS1-2#
kDa
IGF1R
MYC
GAPDH
H3
t
VCaP
siNC
siAS1-1#
siAS1-2#
siAS1-1#+AS1-OV
siAS1-2#+AS1-OV
Relative expression level
MYC
u
VCaP
siRNA-AS1
NC
1#
2#
1#
2#
Empty vector
IGF1R-AS1
kDa
MYC
GAPDH
H3
v
LNCaP
Vector Control
IGF1R-AS1 OV
Relative mRNA level
MYC
w
LNCaP
Vec
OV
kDa
MYC
GAPDH
H3
x
VCaP
siNC
siAS1-1#
siAS1-2#
Relative expression level
CBX3
CDK4
DDX18
MCM4
MCM5
PA2G4
CLNS1A
TXNL4A
MYC active targets
MYC
repressive targets

Supplementary Figure 3. Effects of *IGF1R-AS1* knockdown on cell proliferation, and MYC target gene regulation in prostate cancer cells. **(a)** Pie chart showing prostate tumor samples harboring the *IGF1R-AS1* feature junction were more abundant in metastatic tumors than in localized tumors. **(b)** qPCR analysis of *IGF1R-AS1* expression in VCaP, 22RV1, and H727 cells after *IGF1R-AS1* knockdown with two different siRNAs (n=3 biological replicates). Unpaired Student's t-test, two-sided. p*** (VCaP, siNC vs siAS1-1#)=0.0001, p***(22RV1, siNC vs siAS1-1#)=0.0003, p***(22RV1, siNC vs siAS1-2#)=0.0004, p**(H727, siNC vs siAS1-1#)=0.0012, p***(H727, siNC vs siAS1-2#)=0.0009, p****<0.0001. **(c)** Relative cell growth rates over time in VCaP (n=3 biological replicates), 22RV1 (n=4 biological replicates), and H727 (n=6 biological replicates) cells upon *IGF1R-AS1* knockdown. Unpaired Student's t-test, two-sided. p***(VCaP, siNC vs siAS1-1#, day 5)=0.0001, p**(VCaP, siNC vs siAS1-2#, day 5)=0.0048, p****(22RV1, day 5)<0.0001, p**(H727, siNC vs siAS1-1#, day 4)=0.0012, p*(H727, siNC vs siAS1-2#, day 4)=0.0307. **(d)** 22RV1 cell migration and invasion after *IGF1R-AS1* knockdown (n=3 biological replicates). Scale bar, 100 µm. Unpaired Student's t-test, two-sided. p***(migration, siNC vs siAS1-1#)=0.0005, p***(migration, siNC vs siAS1-2#)=0.0008, p***(invasion, siNC vs siAS1-2#)=0.0003, p****<0.0001. (**e**) qPCR analysis showing rescued expression of *IGF1R-AS1* in VCaP *IGF1R-AS1* knockdown cells transfected with *IGF1R-AS1* overexpression plasmid (n=3 biological replicates). Unpaired Student's t-test, two-sided. p***=0.0004, p****<0.0001. **(f-g)** Relative cell growth rates (f, n=4 biological replicates) and cell migration, invasion (g, n=3 biological replicates) in VCaP cells after *IGF1R-AS1* rescued expression in *IGF1R-AS1* knockdown cells. Scale bar in (g), 100 µm. Unpaired Student's t-test, two-sided. p****(f, day 5)<0.0001. p***(g, migration, siNC vs siAS1-1#)=0.0003, p**(g, migration, siNC vs siAS1-2#)=0.0011, p***(g, migration, siAS1-1# vs siAS1-1#+OV)=0.0007, p**(g, migration, siAS1-2# vs siAS1-2#+OV)=0.0093, p**(g, invasion, siNC vs siAS1-1#)=0.0035, p**(g, invasion, siNC vs siAS1-2#)=0.0046, p*(g, invasion, siAS1-1# vs siAS1-1#+OV)=0.0163, p**(g, invasion, siAS1-2# vs siAS1-2#+OV)=0.0074. **(h)** qPCR analysis of *IGF1R-AS1* expression in LNCaP cells after stably infecting with lentivirus expressing *IGF1R-AS1* (n=3 biological replicates). Unpaired Student's t-test, two-sided. p***=0.0002. **(i-j)** Relative cell growth rates (i, n=4 biological replicates) and cell migration, invasion (j, n=3 biological replicates) in LNCaP cells after *IGF1R-AS1* overexpression. Scale bar in (j), 100 µm. Unpaired Student's t-test, two-sided. p**(i, day 5)=0.0037, p**(j, migration)=0.0011, p**(j, invasion)=0.0062. **(k)** qPCR analysis showing stable *IGF1R-AS1* knockdown in 22RV1 cells infected with lentivirus expressing shRNAs targeting *IGF1R-AS1* or negative control shRNA (n=3 biological replicates). Unpaired Student's t-test, two-sided. p****<0.0001. **(l)** Relative cell growth rate of 22RV1 cells after stably knockdown of *IGF1R-AS1* (n=4 biological replicates). Unpaired Student's t-test, two-sided. p**** (day 5)<0.0001. **(m)** Tumor growth curve in xenograft mouse models of 22RV1 cells after *IGF1R-AS1* stable knockdown. n=8 mice per group. Unpaired Student's t-test, two-sided. p**** (shNC vs shAS1-1#, week 3.5)<0.0001, p* (shNC vs shAS1-2#, week 3.5)=0.0120. **(n)** Kaplan–Meier survival plot in xenograft mouse models of 22RV1 cells after *IGF1R-AS1* stable knockdown, based on the weeks needed when tumor size reaches 1000 mm^3. n=8 mice per group. Log Rank test. p***(shNC vs shAS1-1#)=0.0006, p*(shNC vs shAS1-2#)=0.0286. **(o)** GSEA of genes co-expressed with *IGF1R-AS1* in TCGA-PRAD clinical samples reveals significant enrichment of MYC targets and cell cycle-related pathways. **(p-q)** qPCR analysis of expression of *MYC*, *IGF1R*, and *IGF1R* nearby genes, *SYNM* and *TTC23* in 22RV1 (p) and H727 (q) cells after *IGF1R-AS1* knockdown (n=3 biological replicates). Unpaired Student's t-test, two-sided. p***(p, siNC vs siAS1-1#)=0.0005, p***(p, siNC vs siAS1-2#)=0.0003, p***(q, siNC vs siAS1-1#)=0.0002, p****(q, siNC vs siAS1-2#)<0.0001. **(r-s)** Western blotting analysis of IGF1R and MYC protein levels in 22RV1 (r) and H727 (s) cells following *IGF1R-AS1* knockdown. **(t-u)** qPCR analysis (t, n=3 biological replicates) and western blotting analysis (u) of MYC expression in VCaP *IGF1R-AS1* knockdown cells transfected with *IGF1R-AS1* overexpression plasmid. Unpaired Student's t-test, two-sided. p**(t, siNC vs siAS1-1#)=0.0017, p**(t, siNC vs siAS1-2#)=0.0048, p***(t, siAS1-1# vs siAS1-1#+OV)=0.0005, p**(t, siAS1-2# vs siAS1-2#+OV)=0.0050. **(v-w)** qPCR analysis (v, n=3 biological replicates) and western blotting analysis (w) of MYC expression in LNCaP cells after stably infecting with lentivirus expressing *IGF1R-AS1*. Unpaired Student's t-test, two-sided. p**(v)=0.0064. **(x)** qPCR analysis of MYC active and repressive target genes' expression in VCaP cells upon *IGF1R-AS1* knockdown (n=3 biological replicates). Unpaired Student's t-test, two-sided. p*(*CBX3*, siNC vs siAS1-1#)=0.0196, p**(*CBX3*, siNC vs siAS1-2#)=0.0036, p**(*CDK4*, siNC vs siAS1-1#)=0.0028, p**(*CDK4*, siNC vs siAS1-2#)=0.0017, p*(*DDX18*, siNC vs siAS1-1#)=0.0165, p**(*DDX18*, siNC vs siAS1-2#)=0.0039, p**(*MCM4*, siNC vs siAS1-1#)=0.0069, p**(*MCM4*, siNC vs siAS1-2#)=0.0049, p***(*MCM5*, siNC vs siAS1-1#)=0.0001, p***(*MCM5*, siNC vs siAS1-2#)=0.0001, p**(*PA2G4*, siNC vs siAS1-1#)=0.0015, p**(*PA2G4*, siNC vs siAS1-2#)=0.0020, p***(*CLNS1A*, siNC vs siAS1-1#)=0.0002, p***(*CLNS1A*, siNC vs siAS1-2#)=0.0002, p***(*TXRL4A*, siNC vs siAS1-1#)=0.0002, p*(*TXRL4A*, siNC vs siAS1-2#)=0.0141. Data in (b-m, p-q, t, v, and x) are shown as mean ±

SEM. Experiments in (r-s, u, and w) were biologically repeated three times. Source data of this Figure are provided as Source data file.

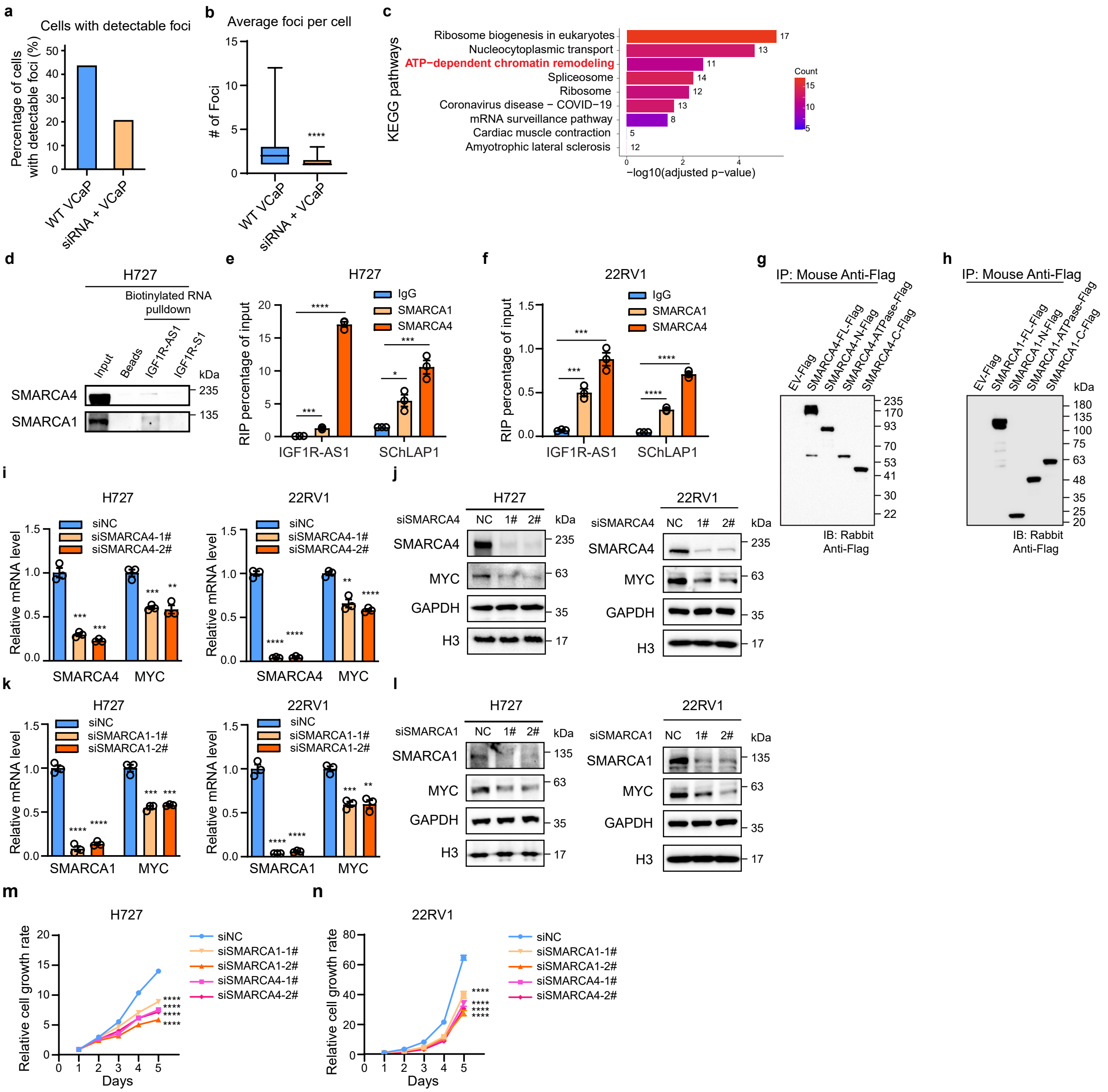
a
Cells with detectable foci
Percentage of cells with detectable foci (%)
WT VCaP
siRNA + VCaP
b
Average foci per cell
# of Foci
****
WT VCaP
siRNA + VCaP
c
KEGG pathways
Ribosome biogenesis in eukaryotes
Nucleocytoplasmic transport
ATP−dependent chromatin remodeling
Spliceosome
Ribosome
Coronavirus disease − COVID−19
mRNA surveillance pathway
Cardiac muscle contraction
Amyotrophic lateral sclerosis
Count
−log10(adjusted p−value)
d
H727
Biotinylated RNA pulldown
Input
Beads
IGF1R-AS1
IGF1R-S1
kDa
SMARCA4
SMARCA1
e
H727
IgG
SMARCA1
SMARCA4
RIP percentage of input
IGF1R-AS1
SChLAP1
f
22RV1
IgG
SMARCA1
SMARCA4
RIP percentage of input
IGF1R-AS1
SChLAP1
g
IP: Mouse Anti-Flag
EV-Flag
SMARCA4-FL-Flag
SMARCA4-N-Flag
SMARCA4-ATPase-Flag
SMARCA4-C-Flag
IB: Rabbit Anti-Flag
h
IP: Mouse Anti-Flag
EV-Flag
SMARCA1-FL-Flag
SMARCA1-N-Flag
SMARCA1-ATPase-Flag
SMARCA1-C-Flag
kDa
IB: Rabbit Anti-Flag
i
H727
22RV1
siNC
siSMARCA4-1#
siSMARCA4-2#
Relative mRNA level
SMARCA4
MYC
j
H727
22RV1
siSMARCA4
NC
1#
2#
kDa
SMARCA4
MYC
GAPDH
H3
k
H727
22RV1
siNC
siSMARCA1-1#
siSMARCA1-2#
Relative mRNA level
SMARCA1
MYC
l
H727
22RV1
siSMARCA1
NC
1#
2#
kDa
SMARCA1
MYC
GAPDH
H3
m
H727
siNC
siSMARCA1-1#
siSMARCA1-2#
siSMARCA4-1#
siSMARCA4-2#
Relative cell growth rate
Days
n
22RV1
siNC
siSMARCA1-1#
siSMARCA1-2#
siSMARCA4-1#
siSMARCA4-2#
Relative cell growth rate
Days

Supplementary Figure 4. Characterization of *IGF1R-AS1* and its interactions with chromatin remodelers SMARCA4 and SMARCA1. (a-b) Quantification of *IGF1R-AS1* FISH signals in VCaP cells. (a) Percentage of cells containing detectable *IGF1R-AS1* foci in WT and siRNA-treated conditions. (b) Average number of *IGF1R-AS1* foci per cell in WT (n=463 cells) and siRNA-treated (n=41 cells) VCaP cells. Boxplot shows the median (center line), interquartile range (box bounds, 25th to 75th percentile), and whiskers extending to 1.5× the interquartile range. Unpaired Student's t-test, two-sided. p****<0.0001. **(c)** KEGG pathway enrichment analysis showing significantly enriched pathways, with -log10(adjusted p-value) and count of genes indicated. Color intensity represents gene count. **(d)** RNA pulldown assay using biotinylated *IGF1R-AS1* followed by Western blot analysis showing interaction with SMARCA1 and SMARCA4 proteins in H727 cells. **(e-f)** RNA immunoprecipitation (RIP) assay in H727 cells (e) and 22RV1 cells (f) showing enrichment of *IGF1R-AS1* and *SChLAP1* (control) with SMARCA1 andSMARCA4 antibodies compared to IgG control (n=3 biological replicates). Unpaired Student's t-test, two-sided. p***(e, *IGF1R-AS1*, SMARCA1 vs IgG)=0.0002, p*(e, *SChLAP1*, SMARCA1 vs IgG)=0.0107, p***(e, *SChLAP1*, SMARCA4 vs IgG)=0.0009, p***(f, *IGF1R-AS1*, SMARCA1 vs IgG)=0.0006, p***(f, *IGF1R-AS1*, SMARCA4 vs IgG)=0.0004, p****<0.0001. **(g-h)** Western blotting of Flag-tagged SMARCA4 (g) and SMARCA1 (h) full-length and truncations enriched by mouse anti-Flag antibody in RIP assays. The primary antibody for western blotting was a rabbit anti-Flag antibody. **(i-j)** qPCR analysis (i, n=3 biological replicates) and western blotting analysis (j) of SMARCA4 and MYC expression following siRNA-mediated knockdown of *SMARCA4* in H727 cells and 22RV1 cells. Unpaired Student's t-test, two-sided. p***(H727, *SMARCA4*, siNC vs siSMARCA4-1#)=0.0003, p***(H727, *SMARCA4*, siNC vs siSMARCA4-2#)=0.0002, p***(H727, *MYC*, siNC vs siSMARCA4-1#)=0.0008, p**(H727, *MYC*, siNC vs siSMARCA4-2#)=0.0026, p**(22RV1, *MYC*, siNC vs siSMARCA4-1#)=0.0025, p****<0.0001. **(k-l)** qPCR analysis (k, n=3 biological replicates) and western blotting analysis (l) of SMARCA1 and Myc expression following siRNA-mediated knockdown of *SMARCA1* in H727 cells and 22RV1 cells. Unpaired Student's t-test, two-sided. p***(H727, *MYC*, siNC vs siSMARCA1-1#)=0.0006, p***(H727, *MYC*, siNC vs siSMARCA1-2#)=0.0005, p***(22RV1, *MYC*, siNC vs siSMARCA1-1#)=0.0007, p**(22RV1, *MYC*, siNC vs siSMARCA1-2#)=0.0019. **(m-n)** Relative cell growth rate in H727 cells (m, n=4 biological replicates) and 22RV1 cells (n, n=6 biological replicates) following *SMARCA1* knockdown. Unpaired Student's t-test, two-sided. p****(day 5)<0.0001. Data in (e-f, i, k, and m-n) are shown as mean ± SEM. Experiments in (d, g-h, j, and l) were biologically repeated three times. Source data of this Figure are provided as Source data file.

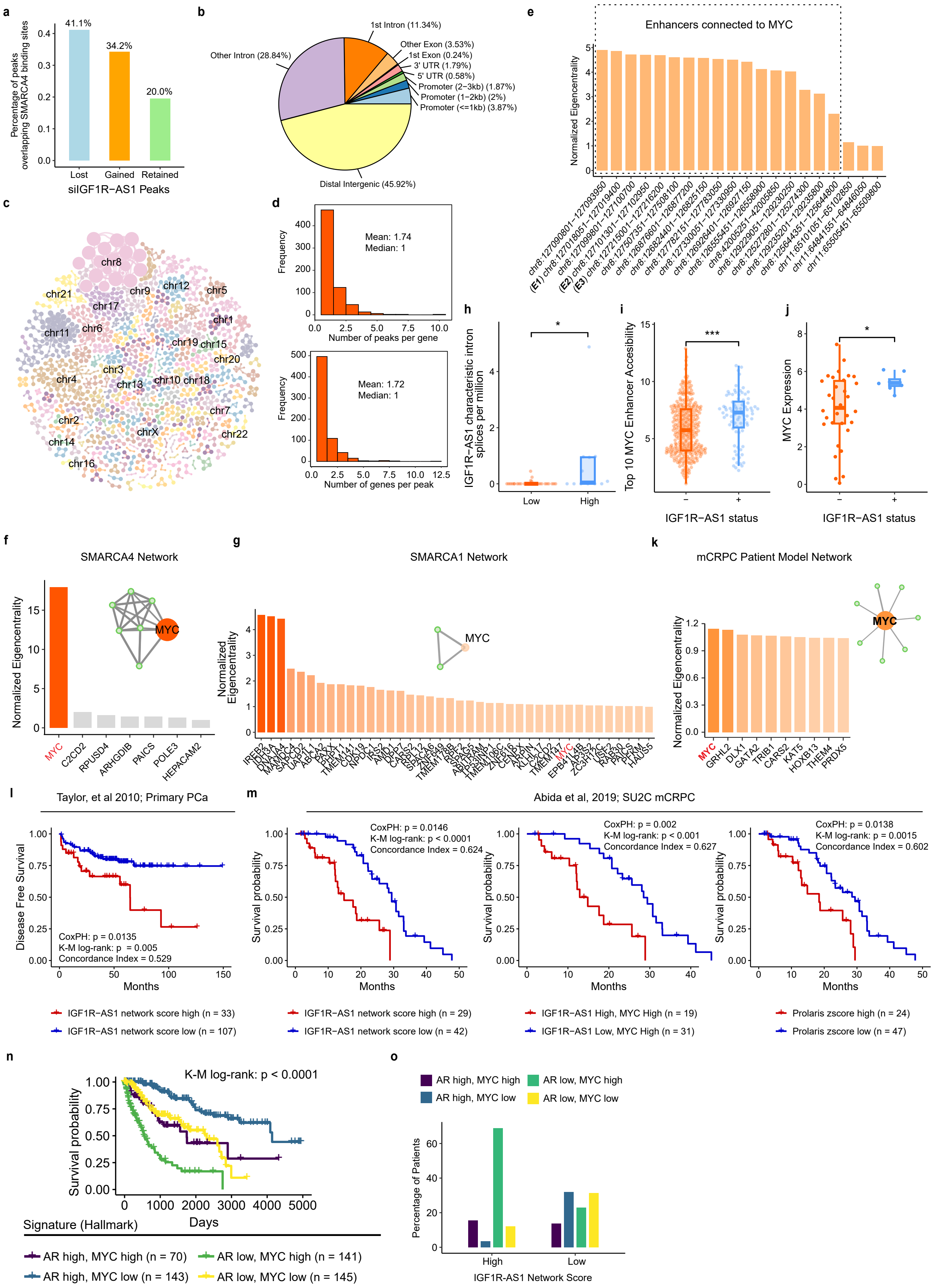

a
Percentage of peaks overlapping SMARCA4 binding sites
41.1%
34.2%
20.0%
Lost
Gained
Retained
siIGF1R−AS1 Peaks
b
1st Intron (11.34%)
Other Exon (3.53%)
1st Exon (0.24%)
3' UTR (1.79%)
5' UTR (0.58%)
Promoter (2−3kb) (1.87%)
Promoter (1−2kb) (2%)
Promoter (<=1kb) (3.87%)
Other Intron (28.84%)
Distal Intergenic (45.92%)
e
Enhancers connected to MYC
Normalized Eigencentrality
chr8:127090801-127093950
(E1) chr8:127018051-127019400
chr8:127099801-127100700
(E2) chr8:127101301-127102950
(E3) chr8:127215001-127216200
chr8:127507351-127508100
chr8:126876601-126877200
chr8:126824401-126825150
chr8:127782151-127783050
chr8:127330051-127330950
chr8:126926401-126927150
chr8:126555451-126558900
chr8:42005251-42005850
chr8:129229051-129230250
chr8:125272801-125274300
chr8:129235201-129235800
chr8:125644351-125644800
chr11:65101051-65102850
chr11:64841551-64846050
chr11:65505451-65509800
c
chr8
chr21
chr9
chr12
chr5
chr17
chr11
chr6
chr1
chr19
chr15
chr20
chr3
chr4
chr13
chr10
chr18
chr7
chr2
chrX
chr22
chr14
chr16
d
Frequency
Mean: 1.74
Median: 1
Number of peaks per gene
Mean: 1.72
Median: 1
Number of genes per peak
h
IGF1R−AS1 characteristic intron splices per million
Low
High
i
Top 10 MYC Enhancer Accesibility
IGF1R−AS1 status
j
MYC Expression
IGF1R−AS1 status
f
SMARCA4 Network
Normalized Eigencentrality
MYC
MYC
C2CD2
RPUSD4
ARHGDIB
PAICS
POLE3
HEPACAM2
g
SMARCA1 Network
Normalized Eigencentrality
MYC
k
mCRPC Patient Model Network
Normalized Eigencentrality
MYC
MYC
GRHL2
DLX1
GATA2
TRIB1
CARS2
KAT5
HOXB13
THEM4
PRDX5
l
Taylor, et al 2010; Primary PCa
Disease Free Survival
CoxPH: p = 0.0135
K-M log-rank: p = 0.005
Concordance Index = 0.529
Months
IGF1R−AS1 network score high (n = 33)
IGF1R−AS1 network score low (n = 107)
m
Abida et al, 2019; SU2C mCRPC
Survival probability
CoxPH: p = 0.0146
K-M log-rank: p < 0.0001
Concordance Index = 0.624
Months
IGF1R−AS1 network score high (n = 29)
IGF1R−AS1 network score low (n = 42)
CoxPH: p = 0.002
K-M log-rank: p < 0.001
Concordance Index = 0.627
IGF1R−AS1 High, MYC High (n = 19)
IGF1R−AS1 Low, MYC High (n = 31)
CoxPH: p = 0.0138
K-M log-rank: p = 0.0015
Concordance Index = 0.602
Prolaris zscore high (n = 24)
Prolaris zscore low (n = 47)
n
K-M log-rank: p < 0.0001
Survival probability
Days
Signature (Hallmark)
AR high, MYC high (n = 70)
AR low, MYC high (n = 141)
AR high, MYC low (n = 143)
AR low, MYC low (n = 145)
o
AR high, MYC high
AR low, MYC high
AR high, MYC low
AR low, MYC low
Percentage of Patients
High
Low
IGF1R-AS1 Network Score

Supplementary Figure 5. *IGF1R-AS1* regulates SWI/SNF-dependent gene regulatory networks. (a) Percentage of ATAC-seq peaks that were lost, gained, or retained overlapping with SMARCA4 binding sites after *IGF1R-AS1* knockdown in VCaP cells. **(b)** Distribution of ATAC-seq peaks across annotated genomic regions. Peaks affected by *IGF1R-AS1* are predominantly localized within enhancer-associated regions, particularly in distal intergenic and intronic regions. **(c)** Genome wide enhancer-promoter interactions regulated by *IGF1R-AS1*. **(d)** Distribution of enhancer-promoter links, quantified per peak (left) and per gene (right), based on the inferred enhancer-promoter interaction network. **(e)** Top-ranked enhancers based on eigencentrality scores, with MYC-associated enhancers highlighted. Three key enhancers are labeled as E1, E2, and E3. **(f-g)** SMARCA4-regulated (f) and SMARCA1-regulated (g) enhancer-promoter interaction network inferred from RNA-seq and ATAC-seq data following *SMARCA4* or *SMARCA1* knockdown in VCaP cells. Bar plot displaying top-ranked genes by eigencentrality scores and visualization of the enhancer-promoter interaction network. **(h)** *IGF1R-AS1* expression levels, assessed via splice junction counts in its first intron, compared between prostate cancer samples with low (n=27 samples) and high (n=13 samples) *MYC* enhancer activity. Boxplot shows the median (center line), interquartile range (box bounds, 25th to 75th percentile), and whiskers extending to 1.5× the interquartile range. Unpaired Wilcoxon rank sum test, two-sided. p*=0.0176. **(i-j)** *IGF1R-AS1*-positive models show increased *MYC* enhancer accessibility (i, n=448 samples in (-), and n=112 samples in (+)) and higher *MYC* expression (j, n=32 samples in (-), and n=8 samples in (+)). Boxplot shows the median (center line), interquartile range (box bounds, 25th to 75th percentile), and whiskers extending to 1.5× the interquartile range. Unpaired Wilcoxon rank sum test, two-sided. p***(i)=0.0002, p*(j)=0.0357. **(k)** *IGF1R-AS1* regulatory network constructed from 40 prostate cancer models, including cell lines, patient-derived xenografts, and mCRPC organoids, highlighting *MYC* as the hub gene with the highest connectivity to enhancers. Left: Bar plot of top-ranked genes by eigencentrality scores. Right: Enhancer-promoter interaction network visualization. **(l)** Kaplan-Meier disease-free survival analysis in primary prostate cancer cohort (Taylor et al., 2010)[1] stratified by *IGF1R-AS1* network score. n=140 patients. **(m)** Kaplan-Meier overall survival analysis in the SU2C mCRPC cohort (Abida et al., 2019)[2]. Left: stratified by *IGF1R-AS1* network score; n=71 patients. Middle: stratified first by MYC Hallmark signature score, then stratified by *IGF1R-AS1* network score in MYC high patients; n=50 patients. Right: stratified by z-score of Prolaris cell-cycle markers; n=71 patients.**(n)** Kaplan-Meier survival analysis of mCRPC patients from Tempus cohort (n=499 patients) stratified by MYC and AR pathway activity levels using optimal cutpoints. **(o)** Percentage of patients with AR high/low and MYC high/low for high or low *IGF1R-AS1* network scores based on Figure 5g. Source data of this Figure are provided as Source data file.

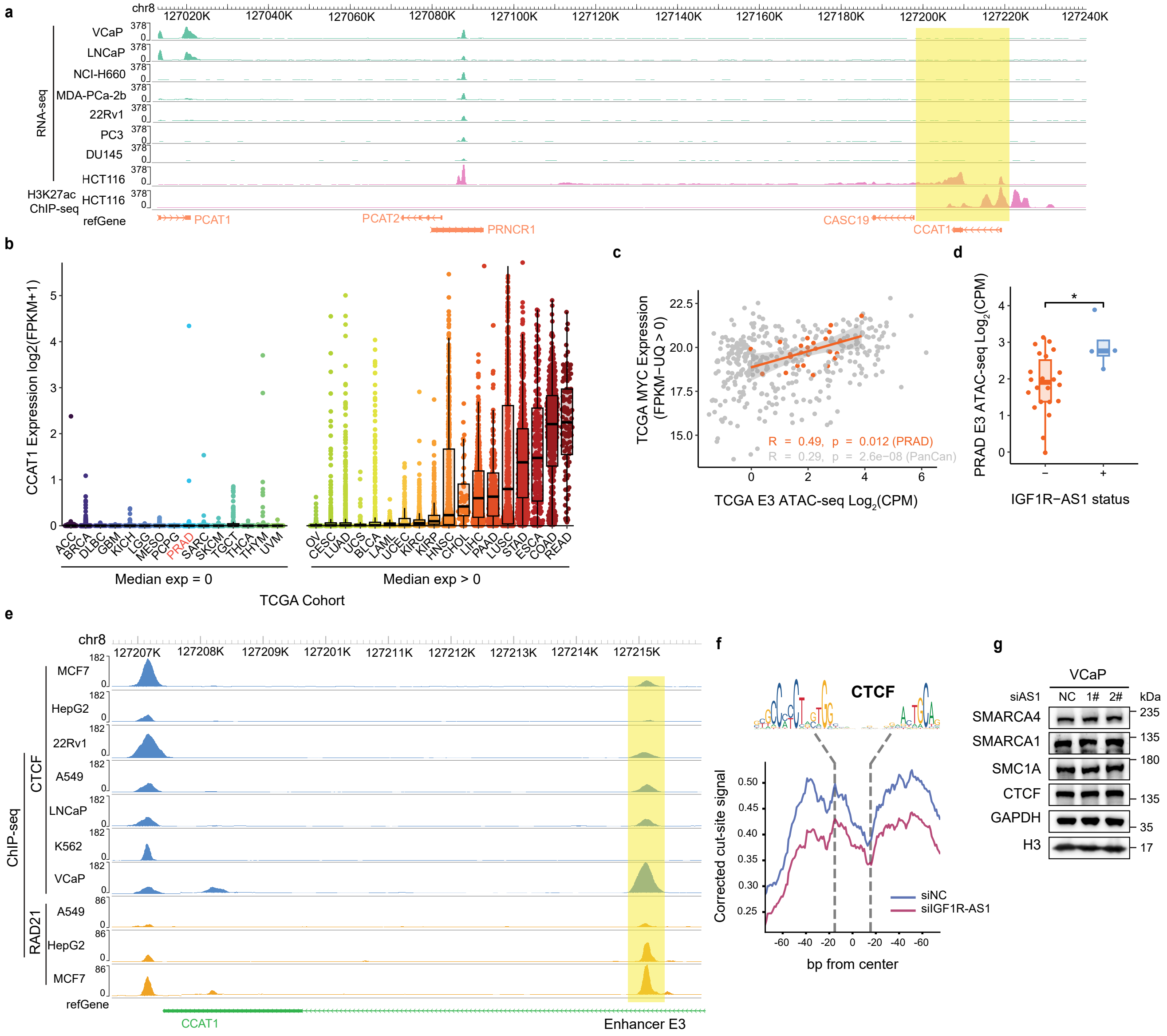

a
chr8
127020K
127040K
127060K
127080K
127100K
127120K
127140K
127160K
127180K
127200K
127220K
127240K
RNA-seq
VCaP
LNCaP
NCI-H660
MDA-PCa-2b
22Rv1
PC3
DU145
HCT116
H3K27ac ChIP-seq
HCT116
refGene
PCAT1
PCAT2
PRNCR1
CASC19
CCAT1
b
CCAT1 Expression log2(FPKM+1)
ACC BRCA DLBC GBM KICH LGG MESO PCPG PRAD SARC SKCM TGCT THCA THYM UVM
OV CESC LUAD UCS BLCA LAML UCEC KIRC KIRP HNSC CHOL LIHC PAAD LUSC STAD ESCA COAD READ
Median exp = 0
Median exp > 0
TCGA Cohort
c
TCGA MYC Expression (FPKM−UQ > 0)
TCGA E3 ATAC-seq Log2(CPM)
R = 0.49, p = 0.012 (PRAD)
R = 0.29, p = 2.6e−08 (PanCan)
d
PRAD E3 ATAC-seq Log2(CPM)
IGF1R−AS1 status
*
e
chr8
127207K
127208K
127209K
127201K
127211K
127212K
127213K
127214K
127215K
ChIP-seq
CTCF
MCF7
HepG2
22Rv1
A549
LNCaP
K562
VCaP
RAD21
A549
HepG2
MCF7
refGene
CCAT1
Enhancer E3
f
CTCF
Corrected cut-site signal
siNC
siIGF1R-AS1
bp from center
g
VCaP
siAS1
NC
1#
2#
kDa
SMARCA4
SMARCA1
SMC1A
CTCF
GAPDH
H3
235
135
180
135
35
17

Supplementary Figure 6. *CCAT1* expression patterns in cancer cell lines and tumor tissues and its regulatory elements. (a) Genome browser tracks display RNA-seq coverage at the *CCAT1* locus and flanking genes across prostate cancer cell lines (VCaP, LNCaP, NCI-H660, MDA-PCa-2b, 22Rv1, PC3, and DU145), with HCT116 colon cancer cells (RNA-seq and H3K27ac ChIP-seq) included as a positive control. **(b)** Expression of *CCAT1* across diverse human tumor tissue samples from TCGA study. Cancer types are grouped based on median expression levels (left panel: median expression = 0; right panel: median expression > 0). **(c)** Scatter plot showing the relationship between the TCGA E3 enhancer signal, measured as log2 normalized counts per million (CPM) of ATAC-seq read counts, and MYC expression across the TCGA Pan-Cancer dataset (gray, n=349 patient samples) and the PRAD (Prostate Adenocarcinoma) subset (orange, n=26 patient samples). Simple linear regression, two-sided. **(d)** Box plot illustrating the E3 enhancer signal in prostate cancer (TCGA-PRAD) samples based on *IGF1R-AS1* status (positive, n=4 sample number vs negative, n=22 sample number). Samples with expressed *IGF1R-AS1* exhibit a significantly higher E3 enhancer signal than those with negative status. Boxplot shows the median (center line), interquartile range (box bounds, 25th to 75th percentile), and whiskers extending to 1.5× the interquartile range. Unpaired Student's t-test, two-sided, *p=0.0253. **(e)** ChIP-seq binding profiles of chromatin regulators (CTCF and RAD21) at the *CCAT1* genomic locus across multiple cancer cell lines. The bottom panel shows the genomic coordinates and gene structure of *CCAT1* on chromosome 8. ChIP-seq signals for CTCF (blue) and RAD21 (orange), are shown for different cell lines as indicated. **(f)** Aggregated ATAC-seq footprint analysis across all CTCF-bound motifs shows *IGF1R-AS1* depletion globally reduces CTCF binding in VCaP cells. (**g**) Western blotting analysis of SMARCA1, SMARCA4, SMC1A, and CTCF expression in VCaP cells after knocking down *IGF1R-AS1*. Experiments in (g) were biologically repeated three times. Source data of this Figure are provided as Source data file.

Supplementary Table 1. IGF1R-AS1 isoforms.

Transcript ID	Chromosome	Start	End	Strand	# of exons	exon positions	cell line
V1,H1	chr15	98839513	98843895	-	2	98839513-98839896; 98843674-98843895	VCaP, H727
V2,H2	chr15	98831668	98843895	-	3	98831668-98831959; 98839790-98839896; 98843674-98843895	VCaP, H727
V3,H4	chr15	98830921	98843895	-	3	98830921-98831563; 98839790-98839896; 98843674-98843895	VCaP, H727
V4	chr15	98838140	98843895	-	2	98838140-98839896; 98843674-98843895	VCaP
V5	chr15	98838140	98843895	-	3	98838140-98838281; 98839790-98839896 ;98843674-98843895	VCaP
V6	chr15	98831208	98843895	-	3	98831208-98831563; 98839790-98839896; 98843674-98843895	VCaP
V7	chr15	98830624	98843895	-	3	98830624-98831563; 98839790-98839896; 98843674-98843895	VCaP
V8	chr15	98838036	98843895	-	2	98838036-98839896; 98843674-98843895	VCaP
V9,H5	chr15	98839513	98843895	-	3	98839513-98839896; 98843308-98843479; 98843674-98843895	VCaP, H727
V10	chr15	98839513	98843895	-	3	98839513-98839896; 98841730-98841946; 98843674-98843895	VCaP
H3	chr15	98838051	98843895	-	3	98838051-98838281; 98839790-98839896; 98843674-98843895	H727
H6	chr15	98831670	98843895	-	4	98831670-98831959; 98839790-98839896; 98843308-98843479; 98843674-98843895	H727
H7	chr15	98831196	98843895	-	3	98831196-98831563; 98839790-98839896; 98843674-98843895	H727
H8	chr15	98831668	98843895	-	4	98831668-98831959; 98838252-98838281; 98839790-98839896; 98843674-98843895	H727
H9	chr15	98838138	98843895	-	4	98838138-98838281; 98839790-98839896; 98843308-98843479; 98843674-98843895	H727
H10	chr15	98838140	98843895	-	3	98838140-98838281; 98839433-98839896; 98843674-98843895	H727

Supplementary References

1 Taylor, B. S. *et al*. Integrative genomic profiling of human prostate cancer. *Cancer Cell* **18**, 11-22 (2010). https://doi.org/10.1016/j.ccr.2010.05.026

2 Abida, W. *et al*. Genomic correlates of clinical outcome in advanced prostate cancer. *Proc Natl Acad Sci U S A* **116**, 11428-11436 (2019). https://doi.org/10.1073/pnas.1902651116
