# Supplementary material for: Tumor-specific lncRNA IGF1R-AS1 trans-regulates chromatin interactions associated with oncogenic MYC signaling
Source: Nat Commun. 2026 Mar 19;17:4171. doi: 10.1038/s41467-026-70814-4 (PMC13153188; doi:10.1038/s41467-026-70814-4)
Supplement: Supplementary file 2 — Description of Additional Supplementary Information [file 41467_2026_70814_MOESM2_ESM.pdf]

Description of Additional Supplementary Information

File name: Supplementary Data 1.

Description: The list of lncRNAs identified in SU2C cohort.

File name: Supplementary Data 2.

Description: The list of proteins identified by mass spectrometry analysis of *IGF1R-AS1* RNA pulldown in VCaP cells.

File name: Supplementary Data 3.

Description: short hairpin RNA (shRNA), Small interfering RNA (siRNA) Oligo sequences.

File name: Supplementary Data 4.

Description: Primer sequences for qRT-PCR, ChIP-qPCR, PCR clone and *in vitro* RNA transcription.
