# Supplementary material for: Tumor-specific lncRNA IGF1R-AS1 trans-regulates chromatin interactions associated with oncogenic MYC signaling
Source: Nat Commun. 2026 Mar 19;17:4171. doi: 10.1038/s41467-026-70814-4 (PMC13153188; doi:10.1038/s41467-026-70814-4)
Supplement: Supplementary file 4 — Reporting Summary [file 41467_2026_70814_MOESM4_ESM.pdf]

nature portfolio

Corresponding author(s): Rendong Yang, Qi Cao, Scott M. Dehm

Last updated by author(s): Feb 13, 2026

Reporting Summary

Nature Portfolio wishes to improve the reproducibility of the work that we publish. This form provides structure for consistency and transparency in reporting. For further information on Nature Portfolio policies, see our Editorial Policies and the Editorial Policy Checklist.

Statistics

For all statistical analyses, confirm that the following items are present in the figure legend, table legend, main text, or Methods section.

n/a	Confirmed	
☐	☒	The exact sample size (n) for each experimental group/condition, given as a discrete number and unit of measurement
☐	☒	A statement on whether measurements were taken from distinct samples or whether the same sample was measured repeatedly
☐	☒	The statistical test(s) used AND whether they are one- or two-sided<br>*Only common tests should be described solely by name; describe more complex techniques in the Methods section.*
☐	☒	A description of all covariates tested
☐	☒	A description of any assumptions or corrections, such as tests of normality and adjustment for multiple comparisons
☐	☒	A full description of the statistical parameters including central tendency (e.g. means) or other basic estimates (e.g. regression coefficient) AND variation (e.g. standard deviation) or associated estimates of uncertainty (e.g. confidence intervals)
☐	☒	For null hypothesis testing, the test statistic (e.g. F, t, r) with confidence intervals, effect sizes, degrees of freedom and P value noted<br>*Give P values as exact values whenever suitable.*
☒	☐	For Bayesian analysis, information on the choice of priors and Markov chain Monte Carlo settings
☒	☐	For hierarchical and complex designs, identification of the appropriate level for tests and full reporting of outcomes
☐	☒	Estimates of effect sizes (e.g. Cohen's d, Pearson's r), indicating how they were calculated

Our web collection on statistics for biologists contains articles on many of the points above.

Software and code

Policy information about availability of computer code

Data collection	Not applicable.
Data analysis	The source codes for novel lncRNA identification and gene-peak regulatory network construction are available at https://github.com/ylab-hi/trans_lncRNA<br>FastQC (version 0.11.8)<br>HISAT2 (version 2.2.1)<br>StringTie (version 1.3.6)<br>BWA-MEM (version 0.7.17)<br>deepTools (version 3.5.1)<br>GFFcompare<br>coding potential prediction: CPAT, CPC2, lncADeep, CNIT, and txCdsPredict<br>FeatureCounts (version 2.0.3)<br>edgeR (version 3.13)<br>ENCODE ChIP-seq transcription factor pipeline (version 2.2.2).<br>Rank Ordering of Super-Enhancers (ROSE) algorithm (https://github.com/stjude/ROSE)<br>CCS tool (version 6.0.0)<br>LIMA (version 2.0.1)<br>ISOSEQ3 (version 3.4.0)<br>minimap2 (version 2.17)<br>SQANTI3 (version 4.0)

RegTools (version 0.4.0)
ENCODE ATACseq pipeline (version 2.2.1)
Cutadapt (version1.9.1)
Bowtie2(version 2.2.6)
SAMtools (version 1.7)
PICARD MarkDuplicates (version 1.26)
MACS2 (version 2.1.0)
csaw R package (version 1.32)
pairtools (version 1.1.3)
juicer (version 1.6)
HiCExplorer (version 3.7)

For manuscripts utilizing custom algorithms or software that are central to the research but not yet described in published literature, software must be made available to editors and reviewers. We strongly encourage code deposition in a community repository (e.g. GitHub). See the Nature Portfolio guidelines for submitting code & software for further information.

Data

Policy information about availability of data

All manuscripts must include a data availability statement. This statement should provide the following information, where applicable:

- Accession codes, unique identifiers, or web links for publicly available datasets
- A description of any restrictions on data availability
- For clinical datasets or third party data, please ensure that the statement adheres to our policy

Data availability
Data generated in this study: Raw and processed data generated in this study have been deposited in the GEO repository (https://www.ncbi.nlm.nih.gov/geo/) under accession number GSE208745. This includes RNA-seq data for IGF1R-AS1, SMARCA1, and SMARCA4 knockdown experiments with controls in VCaP cells, ATAC-seq data for IGF1R-AS1 knockdown with controls, SMARCA1 knockdown with controls in VCaP cells, CTCF HiChIP data for IGF1R-AS1 knockdown with controls in VCaP cells, and PacBio Iso-seq data for IGF1R-AS1 3′ RACE products in VCaP and H727 cells. The mass spectrometry proteomics data are available via ProteomeXchange (http://www.proteomexchange.org/) under accession number PXD074272. The remaining data are available within the Article, Supplementary Information or Source Data file. Source Data are provided with this paper.

Third-Party/Restricted Data: The RNA-seq and associated de-identified clinical annotation data from 499 CRPC patients used in this study were obtained from Tempus AI, Inc. under a research collaboration agreement. These data constitute restricted-access third-party clinical data and were not generated as part of the current study. Due to patient privacy considerations, institutional review board requirements, and commercial data-use agreements, individual-level raw data cannot be made publicly available. Access to the Tempus data may be granted to qualified academic or clinical researchers subject to approval by Tempus AI, Inc. and execution of an appropriate data-use agreement. The authors do not have the authority to grant access to these data.

Publicly Available Repositories:
• SU2C cohort: PolyA-enriched RNA-seq data from 101 metastatic prostate cancer samples, available at dbGaP (https://dbgap.ncbi.nlm.nih.gov/home/) (accession: phs000915.v2.p2).
• TCGA cohort: Normalized ATAC-seq read counts and normalized RNA-seq gene expression values (FPKM-UQ) were obtained from the Xena Browser (https://xenabrowser.net/datapages/).
• Cell line RNA-seq:
o VCaP, MDA-PCa-2b, 22RV1, LNCaP, DU145, NCI-H660, and PC3 (NCBI SRA (https://www.ncbi.nlm.nih.gov/sra) under accessions: SRX5417211, SRX5414821, SRX5414881, SRX5414853, SRX5414453, SRX5414893, SRX5414759).
o H727 cells treated with JQ1 and DMSO were obtained through prior communication with study authors.
o RNA-seq for VCaP with DMSO (24h) and AU-15330 (4h) (NCBI SRA under accessions: SRX10515156, SRX10515157, SRX10515158, SRX10515159).
o Nanopore direct RNA-seq for VCaP (NCBI SRA under accession: SRX26188102)
• Histone ChIP-seq:
o H3K4me3 data for LNCaP, K562, HepG2, HeLa-S3, and GM12878 (ENCODE project (https://www.encodeproject.org/) under accessions: ENCSR000DWF, ENCSR668LDD, ENCSR575RRX, ENCSR000AOF, ENCSR057BWO).
o H3K27ac data for VCaP (ENCODE project under accession: ENCSR597ULV).
o PolII data for VCaP (NCBI SRA under accession: SRX471863).
o H3K4me3 data for VCaP (NCBI SRA under accession: SRX022554).
• TF ChIP-seq, HiChIP and and ATAC-seq:
o BRD4 and PolII data for VCaP (GEO under accession: GSE148358).
o SMARCA4 data for VCaP with EtOH treatment, siSMARCA4 ATAC-seq with EtOH treatment, and corresponding controls (GEO under accession: GSE136016).
o SMARCA4 data for 22RV1 and LNCaP (NCBI SRA under accessions: SRX4193367, SRX2545045).
o ATAC-seq for VCaP with DMSO treatment (24h) and AU-15330 treatment (4h) (NCBI SRA under accessions: SRX10525411, SRX10525413; peaks: GEO under accession: GSE171584).
o H3K27ac and CTCF HiChIP-seq and ChIP-seq for VCaP with AU-15330 (4h) and DMSO (24h) (GEO under accession: GSE171591).
• ChIA-PET: PolII data for VCaP, LNCaP, and DU145 (GEO under accession: GSE121020).
• CRPC model data: RNA-seq and ATAC-seq (GEO under accession: GSE199190).
• BigWig files: CTCF and RAD21 ChIP-seq data (ENCODE project under accessions: ENCFF507CRU, ENCFF704QYE, ENCFF539QXW, ENCFF336UPT, ENCFF083AEY, ENCFF341HKN, ENCFF543QNU, ENCFF867GSQ, ENCFF653EFX, ENCFF775EKJ).

Research involving human participants, their data, or biological material

Policy information about studies with human participants or human data. See also policy information about sex, gender (identity/presentation), and sexual orientation and race, ethnicity and racism.

Reporting on sex and gender	Not applicable
Reporting on race, ethnicity, or other socially relevant groupings	Not applicable
Population characteristics	Not applicable
Recruitment	Not applicable
Ethics oversight	Not applicable

Note that full information on the approval of the study protocol must also be provided in the manuscript.

Field-specific reporting

Please select the one below that is the best fit for your research. If you are not sure, read the appropriate sections before making your selection.

- [x] Life sciences
- [ ] Behavioural & social sciences
- [ ] Ecological, evolutionary & environmental sciences

For a reference copy of the document with all sections, see nature.com/documents/nr-reporting-summary-flat.pdf

Life sciences study design

All studies must disclose on these points even when the disclosure is negative.

Sample size	We analyzed datasets from the public available databases and generated our own data as well. No statistical methods were used to predetermine sample sizes, but our sample sizes are similar to those reported in previous studies. The results were reproducible and conducted with established internal controls. When feasible, experiments were repeated two or more times and yielded similar results.
Data exclusions	No data were excluded from the analysis.
Replication	For bench experiments, we replicated our biological findings for at least 2 times. For RNA-seq and mass spectrometry experiments, at least 2 biological replicates were performed. For computational analysis, random sampling approaches were used for some analysis. For each analysis, we presented the sample size, mean, and p-value when applicable. And we ensured that the findings were reproducible.
Randomization	Mice were injected with cancer cell and randomized for any treatment.
Blinding	For bench experiments, the control and treatment samples were processed side by side to remove batch effects. Blinding was not relevant in this study.

Reporting for specific materials, systems and methods

We require information from authors about some types of materials, experimental systems and methods used in many studies. Here, indicate whether each material, system or method listed is relevant to your study. If you are not sure if a list item applies to your research, read the appropriate section before selecting a response.

Materials & experimental systems

n/a	Involved in the study	
[ ]	[x]	Antibodies
[ ]	[x]	Eukaryotic cell lines
[x]	[ ]	Palaeontology and archaeology
[ ]	[x]	Animals and other organisms
[ ]	[x]	Clinical data
[x]	[ ]	Dual use research of concern
[x]	[ ]	Plants

Methods

n/a	Involved in the study	
[x]	[ ]	ChIP-seq
[x]	[ ]	Flow cytometry
[x]	[ ]	MRI-based neuroimaging

Antibodies

Antibodies used	Anti-SMARCA1 (MABE366) and anti-Flag (F1804) were purchased from MilliporeSigma. Anti-GAPDH (sc-47724) was purchased from Santa Cruz Biotechnology. Anti-c-Myc (#18583), anti-Histone 3 (#9715), anti-IGF1R β (#9750), anti-SMARCA1 (#12483), anti-CTCF (#3418), and anti-Flag (#14793) were purchased from Cell Signaling Technology. Anti-BRD4 (#A301-985A100) and anti-SMC1A

	(#A300-055A) were purchased from Bethyl Laboratories. Anti-SMARCA4 (ab110641) was purchased from Abcam. All antibodies were prepared at a 1:1,000 dilution in TBST buffer in Western blot analysis. Goat anti-mouse/rabbit/rat IgG (H+L)-HRP secondary antibody (GenDEPOT, SA002-500, SA001-500, SA006-500, diluted to 1:4000)
Validation	All the antibodies were validated by manufacturer, and the information about the validation can be found on the manufacturer's website through the links below:<br>https://www.cellsignal.com/<br>https://www.fortislife.com/<br>https://www.abcam.com/en-us<br>https://www.sigmaaldrich.com/US/en<br>https://www.scbt.com/<br>https://gendepot.com/

Eukaryotic cell lines

Policy information about cell lines and Sex and Gender in Research

Cell line source(s)	Prostate cancer cell line VCaP, 22RV1, and LNCaP cells, lung cancer cell line H727, and 293T were purchased from the American Type Culture Collection (ATCC).
Authentication	All cell lines were authenticated by short tandem repeat (STR) genotyping and were used within 2 months of continuous culturing.
Mycoplasma contamination	Cell lines were Mycoplasma negative as reported by routine laboratory tests.
Commonly misidentified lines (See ICLAC register)	No commonly misidentified cell lines were used in this study.

Animals and other research organisms

Policy information about studies involving animals; ARRIVE guidelines recommended for reporting animal research, and Sex and Gender in Research

Laboratory animals	CB-17 SCID male mice (strain 236, ages 6-7 weeks) and NOD-SCID NCG male mice (strain 572, ages 5-6 weeks) were purchased from Charles River.
Wild animals	No wild animals were used in the study.
Reporting on sex	Experiments were performed in male mice since we study prostate cancer.
Field-collected samples	The study did not involve field-collected samples.
Ethics oversight	All animal care and experimental procedures were conducted in compliance with institutional and National Institutes of Health (NIH) guidelines, with approval from the University of Minnesota and Northwestern University Institutional Animal Care and Use Committee (IACUC).

Note that full information on the approval of the study protocol must also be provided in the manuscript.

Clinical data

Policy information about clinical studies

All manuscripts should comply with the ICMJE guidelines for publication of clinical research and a completed CONSORT checklist must be included with all submissions.

Clinical trial registration	*Provide the trial registration number from ClinicalTrials.gov or an equivalent agency.*
Study protocol	*Note where the full trial protocol can be accessed OR if not available, explain why.*
Data collection	*Describe the settings and locales of data collection, noting the time periods of recruitment and data collection.*
Outcomes	*Describe how you pre-defined primary and secondary outcome measures and how you assessed these measures.*

Plants

Seed stocks	*Report on the source of all seed stocks or other plant material used. If applicable, state the seed stock centre and catalogue number. If plant specimens were collected from the field, describe the collection location, date and sampling procedures.*
Novel plant genotypes	*Describe the methods by which all novel plant genotypes were produced. This includes those generated by transgenic approaches, gene editing, chemical/radiation-based mutagenesis and hybridization. For transgenic lines, describe the transformation method, the number of independent lines analyzed and the generation upon which experiments were performed. For gene-edited lines, describe the editor used, the endogenous sequence targeted for editing, the targeting guide RNA sequence (if applicable) and how the editor was applied.*
Authentication	*Describe any authentication procedures for each seed stock used or novel genotype generated. Describe any experiments used to assess the effect of a mutation and, where applicable, how potential secondary effects (e.g. second site T-DNA insertions, mosiacism, off-target gene editing) were examined.*
